# MiR-124-3p mediates gastric cancer cell ferroptosis induced by an anti-cancer drug polyphyllin I

**DOI:** 10.3389/fphar.2023.1285799

**Published:** 2023-11-07

**Authors:** Fang Zheng, Jian-Can Bi, Yu-Yan Wei, Yeshu Wang, Qunfang Zhang, Chun-Ling Liang, Jianwei Wu, Zhenhua Dai

**Affiliations:** ^1^ Joint Immunology Program, The Second Affiliated Hospital of Guangzhou University of Chinese Medicine, Guangzhou, Guangdong, China; ^2^ Department of Internal Medicine, The First People’s Hospital of Jingdezhen, Jingdezhen, Jiangxi, China

**Keywords:** antitumor drug, ferroptosis, gastric cancer, MiR-124-3p, polyphyllin I

## Abstract

**Background:** Ferroptosis is an emerging type of regulated cell death and associated with antitumoral therapy, while some microRNAs have been shown to regulate the tumorigenesis and cancer progression. Meanwhile, polyphyllin I (PPI) has exhibited antitumoral effects by promoting cancer cell apoptosis and ferroptosis. However, it is unclear whether PPI induces cancer cell ferroptosis by regulating microRNAs.

**Methods:** We used two gastric cancer cell lines (AGS and MKN-45) to set up a tumor model of the nude mice, which were then treated daily with PPI to measure the cancer growth *in vitro* and *in vivo*. Ferroptosis was measured using immunofluorescence staining and flow cytometric analysis according to levels of intracellular ROS, lipid ROS and ferrous ions. Moreover, NRF2 expression was measured by Western blotting. In some experiments, the mimics or inhibitors of miR-124-3p were used to further confirm its involvement in PPI-induced cancer cell ferroptosis.

**Results:** Here we found that miR-124-3p mediated cancer ferroptosis and tumor repression induced by PPI since PPI increased miR-124-3p expression in gastric cancer cells and promoted their ferroptosis, whereas inhibition of miR-124-3p mostly abolished the effects of PPI on tumor growth, ferroptosis and NRF2 expression. Moreover, miR-124-3p mimics promoted cancer cell ferroptosis by downregulating NRF2 through directly targeting 3′-UTR region of NRF2, confirming a role for miR-124-3p in regulating PPI-induced ferroptosis.

**Conclusion:** PPI exerts its antitumoral effects on the gastric cancer by promoting cell ferroptosis via regulating miR-124-3p. Our findings have clinical implications for cancer chemotherapy.

## Introduction

Ferroptosis is emerging as a new type of programed cell death, characterized by the increased accumulation of iron, the free radical production and lipid peroxidation (Dixon et al., 2012; Jiang et al., 2021; Tang et al., 2021; Fernandez-Garcia et al., 2022). So far, molecular mechanisms responsible for ferroptosis are not fully understood. Early studies have demonstrated that ferroptosis is associated with carcinogenesis and that enhanced cancer ferroptosis results in tumor repression (Hassannia et al., 2019; Hu et al., 2022; Lei et al., 2022). The drugs that cause different types of cancer cell death and suppression of its growth may also help improve the efficiency of radiotherapy and immune-based therapy (Chen et al., 2021; Zhang et al., 2022; Zhao et al., 2022). Thus, seeking new drugs that can promote cancer cell ferroptosis may open a new door to treating various cancers (Liang et al., 2019), especially drug-resistant cancers.

The non-coding RNAs, especially microRNAs, have been shown to regulate gastric cancer development and serve as biomarkers and therapeutic targets for gastric cancer (Shin and Chu, 2014; Kipkeeva et al., 2020). For instance, miR-34 is dysregulated in gastric cancer (Jafari and Abediankenari, 2017), while miR-21 plays a role in the pathogenesis, diagnosis and prognosis (Farasati Far et al., 2023). On the other hand, non-coding RNAs or microRNAs have also been confirmed to affect tumor cell ferroptosis (Balihodzic et al., 2022; Luo et al., 2022; Zuo et al., 2022). Since they regulate cell ferroptosis, ferroptosis-targeted therapies could be developed for treating various cancers (Guo et al., 2022). In particular, miR-124-3p has reportedly been found to inhibit tumor growth and prevent the progression of some cancers (Li et al., 2014; Liu et al., 2018; Wu Q. et al., 2020; Li et al., 2021; You et al., 2021; Zhu et al., 2021). However, it’s unknown if miR-124-3p is involved in inhibition of tumor growth by inducing cell ferroptosis. Thus, it is important to define whether miR-124-3p suppresses gastric cancer via promoting ferroptosis of gastric cancer cells.

Polyphyllin I (PPI) is a natural ingredient that is purified from the root of *Paris polyphylla.* It is proved to be antitumoral against some cancers (Tian et al., 2020). The mechanisms responsible for the effects of PPI on cancers have also been revealed previously. For instance, PPI activated the conventional AMPK signaling to repress non-small cell lung cancer (Wu Y. et al., 2020b; Shen et al., 2021), reduced the angiogenesis in tumors via working on Twist1/VE-cadherin axis, and promoted apoptotic cell death of hepatocellular carcinoma (Shi et al., 2015; Xiao et al., 2018; Zeng et al., 2020). PPI also promoted gastric cancer cell apoptosis by suppressing STAT3 signaling (Han et al., 2020), while helping lung cancer cells overcome their resistance to erlotinib treatment via inhibition of STAT3 (Lou et al., 2017). Moreover, it accelerated cancer cell autophagy and inhibited cell cycle progress by interfering with Akt/mTOR activation (He et al., 2019). We recently found that PPI induced gastric cancer cell ferroptosis (Zheng et al., 2023). However, the mechanisms for this action of PPI remain unclear.

In this study, we examined the potential involvement of miR-124-3p in PPI-induced gastric cancer cell ferroptosis using two types of cancer cell lines (AGS and MKN-45). We demonstrated that PPI increased miR-124-3p expression in both the gastric cancers *in vivo* and AGS/MKN-45 cells *in vitro*. More importantly, we revealed that miR-124-3p participated in PPI-induced cancer cell ferroptosis and suppression of tumor growth, given that its mimics promoted ferroptosis in both cell lines and suppressed their growth *in vitro* and that an inhibitor of miR-124-3p largely reversed PPI-mediated cell ferroptosis and inhibition of the tumor growth *in vivo* or *in vitro*.

## Materials and methods

### Animals

BALB/c nude mice (6–8 weeks old, female, and 18–22 g) were bought from Experimental Animal Center of Guangdong Province (Guangzhou, Guangdong). Mice were kept in a pathogen-free facility with fixed conditions. All animal experiments complied with the national guidelines of China for the care and use of laboratory animals. Experiments involving animals were approved by the Ethics Committee of the Second Affiliated Hospital of Guangzhou University of Chinese Medicine.

### Reagents

Polyphyllin I (PPI) was purchased from Meilun Biotechnology Co., Ltd. (Lot number: MB7074, Dalian, China, purity>98%), while 3-(4,5-Dimethylthiazol-2-yl)-2,5-diphenyltetrazolium bromide (MTT) was bought from Sigma Aldrich (St. Louis, United States). mAb against NRF2 was purchased from Cell Signaling Technology (Danvers, United States). The mimic, an inhibitor of miR-124-3p or its antagonist (Antagomir) was ordered from Ribo Biological Co., Ltd (RiboBio., Guangzhou, Guangdong).

### Tumor animal models

A subcutaneous tumor model of gastric cancer was established by subcutaneously injecting 1 × 10^6^ AGS or 2 × 10^6^ MKN-45 cells near the right axilla of the mice. Seven days after tumor cell inoculation, mice were injected i.p. daily with PPI (*3 mg/kg*, dissolved in 1% DMSO +5% PEG300 + 5% Tween 80 + 89% deionized water), as described in our previous study (Zheng et al., 2023), or control solution with the same solvent. Mice were weighed at day 15, while tumor volumes were measured every 3 days and calculated using a formula: length X width ^2^/2.

### Treatment of mice

To define a role for miR-124-3p in tumor growth, AGS cells (1 × 10^6^/mouse) were injected to BALB/c nude mice. Seven days later, an antagonist of miR-124-3p (Antagomir; 7.5 nmol per mouse) or its negative control (NC) was injected to the tumors twice a week, while mice received daily PPI following the initial injection of Antagomir. 15 days after daily PPI treatment, tumors were finally harvested for further analyses.

### Cell lines and cultures

Human gastric cancer cell line, MKN-45, was obtained from Chinese Academy of Sciences Cell Bank of Type Culture Collection (Shanghai, China), while human cell line AGS was purchased from American Type Culture Collection (ATCC, Manassas, United States). These tumor cells were cultured in RPMI-1640 medium (Gibco, United States), supplemented with 10% (v/v) fetal bovine serum (Hyclone, United States), 100 μg/mL streptomycin and 100 U/mL penicillin (Gibco), in a humidified incubator containing 5% CO2 at 37°C. Cells with passage numbers of 15–20 were generally used for further experimentation.

### Measurement of intracellular ROS and lipid peroxidation

To measure ROS levels, AGS and MKN-45 cells were labeled with a DCFH-DA fluorescent probe (MCE, United States). Briefly, cells were first treated with PPI and stained with DCFH-DA (10 μM, in HBSS) at 37°C for 30 min. The mean fluorescence intensity (MFI) of DCFH-DA was measured using a flow cytometer (ACEA, Novo Quanteon, United States). To determine lipid-ROS level, a C11-BODIPY fluorescent probe (ThermoFisher, United States) was utilized to determine lipid peroxidation. Cells were then incubated in HBSS containing 5 μM C11-BODIPY at 37°C for 30 min and collected for flow cytometric analyses. MFI was utilized to represent lipid-ROS levels.

### Detection of intracellular Fe2+ ions

To measure intracellular Fe2+ ions, fluorescent probes were used to label Fe2^+^ in cells. Briefly, AGS and MKN-45 cells were seeded in 48-well or 6-well plates at a density of 1 × 10^5^ cells/mL and incubated overnight. After the treatment, cells were then incubated in HBSS containing FerroOrange (Dojindo, Japan, concentration: 1 μM) in an incubator at 37°C and 5% CO2 for 30 min. Cells then were observed under an inverted fluorescence microscope. Furthermore, cells were resuspended in PBS for flow cytometric analyses to quantify intracellular Fe2^+^ ion.

### Quantitative RT- PCR

Trizol reagents (Roche, Switzerland) were used to extract total RNA from cells or tumor tissues. The extracted RNA was reversely transcribed into cDNA via miRNA 1st Strand cDNA Synthesis Kit (Vazyme, China). Quantitative real-time PCR was performed on an ABI Quant Studio 7 Flex PCR System (Applied Biosystems, United States) using miRNA Universal SYBR qPCR Master Mix (Vazyme, China) in 20 μL of the mix containing 2 μL of cDNA preparation. Primers for miR-124-3p and U6 were purchased from Ribo Biological Co., Ltd (RiboBio., China). The result was analyzed according to the 2^ΔΔ^ CT method.

### Treatment of cells with a mimic or inhibitor of miR-124-3p

AGS and MKN-45 cells were transfected with the mimics or inhibitors of miR-124-3p and their corresponding negative controls (NC) using the Lipofectamine 3,000 reagents (Invitrogen-ThermoFisher, United States) according to the manufacturer’s instructions. The final concentrations for the transfections of the mimic and inhibitor were 50 nM and 100 nM, respectively. Finally, RNAs and proteins were extracted for further analyses after culture.

### Dual-luciferase reporter assays

The reporter plasmids (pmirGL0) of wild type (WT) based on the predicted binding sites of miR-124-3p and 3′-UTR of NRF2 were synthesized by GeneCopoeia, Inc. (Rockville, MD, United States). The mutant type (Mut) was created without the specific microRNA-binding sequence. Cells were co-transfected with the reporter plasmids and miR-124-3p mimics or its negative control (NC) mimics using the Lipofectamine 3,000 Transfection Reagent Kit (ThemoFisher, United States). The preparation of cell lysates and measurement of luciferase activities were performed using the Luc-Pair™ Duo-Luciferase HS Assay Kit (GeneCopoeia, United States) according to the manufacturer’s instructions. Luciferase activity was normalized with RLuc activity within each sample.

### Additional methods

*In vitro* tumor cell growth assays (MTT) and Western blot assays of NRF2 expression were described exactly in our recent publication (Zheng et al., 2023).

### Statistical analysis

Statistical analyses were done using GraphPad Prism 8.0 software (La Jolla, CA). The data were analyzed using either *t*-test or One-way ANOVA. The statistics were deemed significant when “*p* < 0.05” occurred.

## Results

### PPI upregulates miR-124-3p expression in the gastric cancer, while an increase in miR-124-3p mimics inhibits cancer cell expansion *in vitro* and promotes their ferroptosis

**FIGURE 1:**
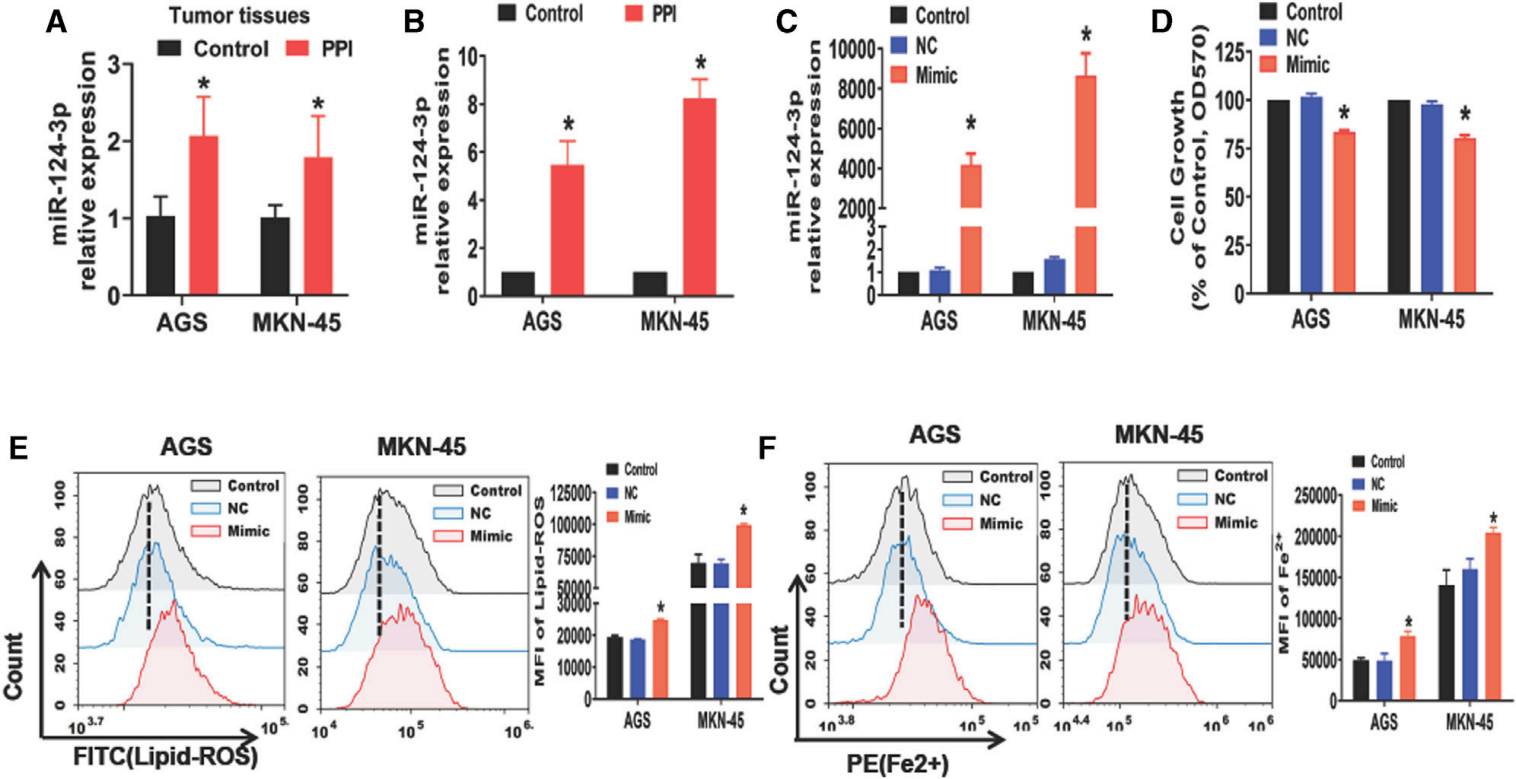
PPI increases miR-124-3p level in gastric cancer cells, while its mimic inhibits their growth and induces ferroptosis. **(A)** miR-124-3p expression in tumors derived from PPI-treated mice was determined using qRT-PCR (n = 6 mice/group per experiment). **(B)** AGS and MKN-45 cells were cultured with PPI (3 μM) for 24h, and their miR-124-3p expression was examined. **(C)** Exogenously increased miR-124-3p expression was confirmed in AGS and MKN-45 cells after addition of miR-124-3p mimics (50 nM) to their culture for 24 h. **(D)** Cell growth was determined using an MTT assay. **(E,F)** Lipid-ROS and Fe^2+^ levels in AGS and MKN-45 cells were determined by flow analysis. The data are shown as Mean ± SD (*p < 0.05).

Previous studies have reported a reduction in miR-124-3p in some types of cancers, resulting in the tumor progression (Wu Q. et al., 2020a; Jin and Zhang, 2020; Li et al., 2021). We therefore asked if miR-124-3p was involved in PPI-induced cancer cell ferroptosis. Here we found that PPI increased miR-124-3p expression in AGS and MKN-45 tumors *in vivo* (Figure 1A) as well as in both cancer cells *in vitro* (Figure 1B). We then applied the mimics of miR-124-3p to both cells via transfection *in vitro* (Figure 1C) and found that the mimics indeed reduced the cancer cell growth (Figure 1D), while increasing intracellular lipid-ROS and Fe^2+^ levels (Figures 1E, F). These findings imply that miR-124-3p represses the gastric tumor by promoting ferroptosis.

### The miR-124-3p directly targets the 3′-UTR region of NRF2 to downregulate NRF2 expression in gastric cancer cells

**FIGURE 2:**
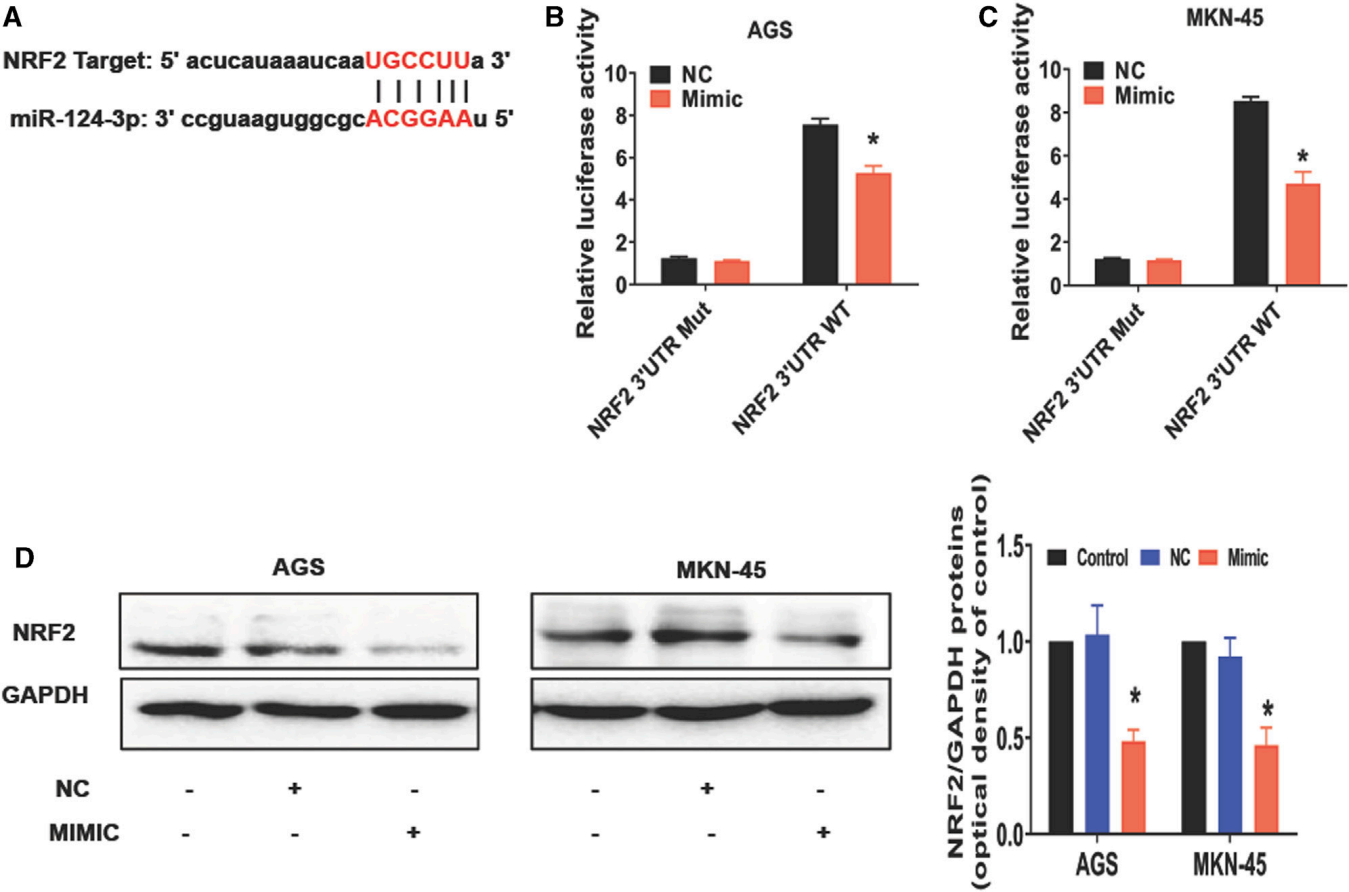
miR-124-3p directly targets 3′UTR of NRF2 to downregulate NRF2 in gastric cancer cells. **(A)** A binding site of miR-124-3p and NRF2 was predicted according to the biological database ENCORI (StarBase database: https://starbase.sysu.edu.cn/). **(B,C)** Shown are luciferase-reporter gene activity, including WT and mutant (Mut) binding sites at the 3′-UTR region of NRF2 mRNA. We transfected AGS and MKN-45 cells with NRF2 3′-UTR-WT or -Mut vectors for 24 h and treated them with miR-124-3p mimics (50 nmol/L) or negative control (NC) for another 24 h. Luciferase activity was determined through Luc-Pair™ Duo-Luciferase HS Assay Kits. **(D)** NRF2 expression was analyzed using Western blotting. Data are shown as mean ± SD (*p < 0.05).

To further understand how miR-124-3p represses the gastric cancer, we analyzed its downstream target genes using the database of bioinformatics (ENCORI). We showed that miR-124-3p exhibited a binding site at the 3′-UTR region of NRF2 (Figure 2A). Therefore, we augmented miR-124-3p expression in AGS and MKN-45 cells by transfecting these cells with its mimics. To confirm the interaction between miR-124-3p and NRF2, we generated 3′-UTR cDNA fragment of NRF2 containing WT and mutant miR-124-3p-binding sites, respectively. As shown in Figures 2B, C, we demonstrated that the interaction of miR-124-3p with WT, but not the mutant (Mut), 3′-UTR cDNA fragment of NRF2 decreased the luciferase activity in both AGS and MKN-45 cells compared to the negative control (NC group).

NRF2 is a transcription factor involved in controlling lipid peroxidation or cell ferroptosis (Dodson et al., 2019). Studies have reported that NRF2 regulates signaling pathways associated with ferroptosis (Dong et al., 2021; Fu et al., 2021; Guan et al., 2022). Using Western blotting to measure ferroptosis-related molecules in cancer cells after exposure to miR-124-3p mimics, we discovered that it inhibited NRF2 expression in both AGS and MKN-45 tumor cells compared with the control or negative control for the mimics (NC) (Figure 2D). These results revealed a direct effect of miR-124-3p on NRF2 expression in gastric cancer cells.

### Inhibition of miR-124-3p reverses PPI-induced ferroptotic cell death of gastric cancer

**FIGURE 3:**
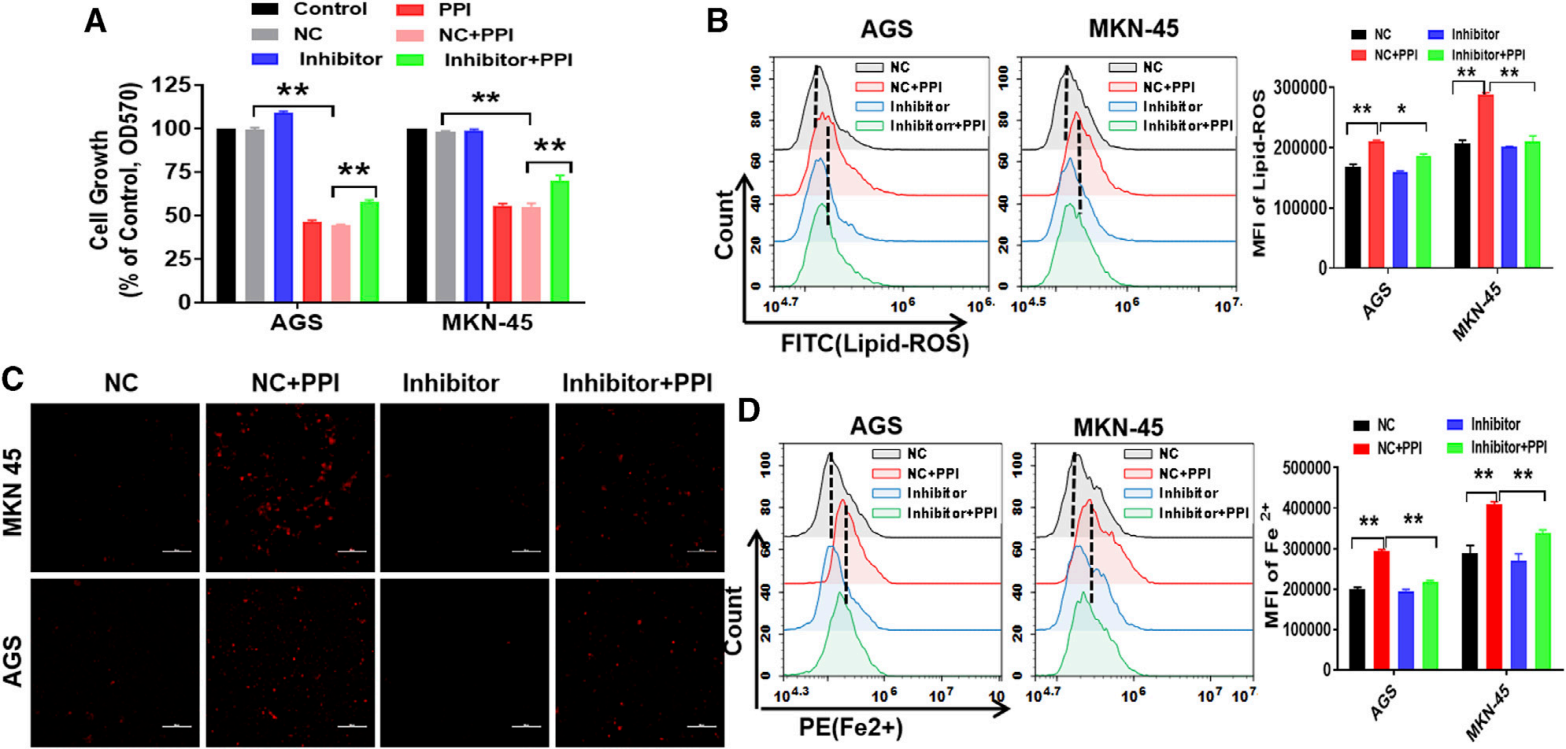
Suppression of miR-124-3p decreases PPI-induced ferroptotic cancer cell death *in vitro*. AGS and MKN-45 tumor cells were first transfected with miR-124-3p inhibitor (100 nM) or the negative control (NC) for 24 h prior to exposure to PPI (3 μM) for 24 h. **(A)** Cell expansion was assessed using MTT; **(B)** Intracellular lipid peroxide (lipid-ROS) was quantified via flow cytometry; **(C,D)** The intracellular levels of ferrous ions (Fe^2+^) were observed via fluorescence microscopy and flow cytometry. The data are shown as mean ± SD (*p < 0.05, **p < 0.01).

To confirm whether miR-124-3p is truely involved in PPI-induced cell ferroptosis, we examined the effects of an inhibitor of miR-124-3p on PPI-mediated inhibition of the tumor cell growth and PPI-induced augmentation of lipid peroxides/ferrous ions in gastric cancer cells. Based on MTT assays, we demonstrated that suppression of miR-124-3p mostly abolished the effects of PPI on the growth of the cancer cells (Figure 3A). According to flow cytometric analysis and immunofluorescence, we found that miR-124-3p inhibitor mostly blocked the lipid peroxidation (Figure 3B) and ferrous ion accumulation induced by PPI (Figures 3C, D).

**FIGURE 4:**
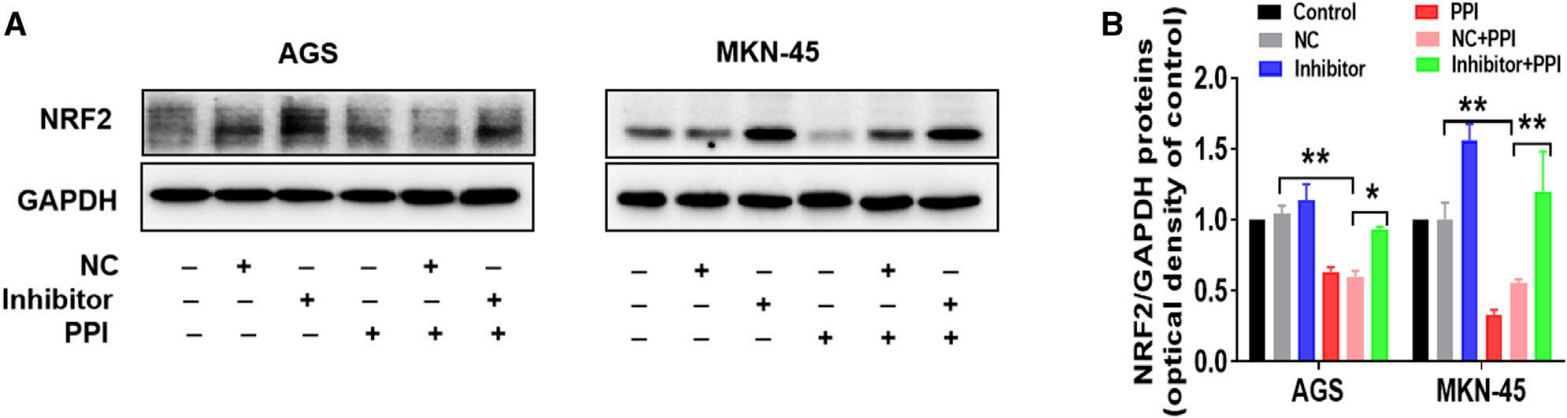
miR-124-3p mediates PPI-induced downregulation of NRF2 in gastric cancer cells. AGS and MKN-45 tumor cells were transfected with miR-124-3p inhibitor (100 nM) or negative control (NC) and then treated with PPI as described in Figure 3. NRF2 protein level in these cells was detected using Western blot assay. Shown is one set of representative images for both tumor cell lines **(A)**. The data are also shown as mean ± SD **(B)** (*p < 0.05, **p < 0.01).

On the other hand, we also observed the effects of an inhibitor of miR-124-3p on NRF2 protein expression. We revealed that the miR-124-3p inhibitor also largely abolished a PPI-induced decrease in NRF2 protein level (Figures 4A, B). These findings suggest that miR-124-3p mediates PPI-induced cancer cell ferroptosis as well as downregulation of NRF2 in the cancer cells.

### Inhibition of miR-124-3p largely reverses the inhibitory effects of PPI on the gastric cancer growth *in vivo*

**FIGURE 5:**
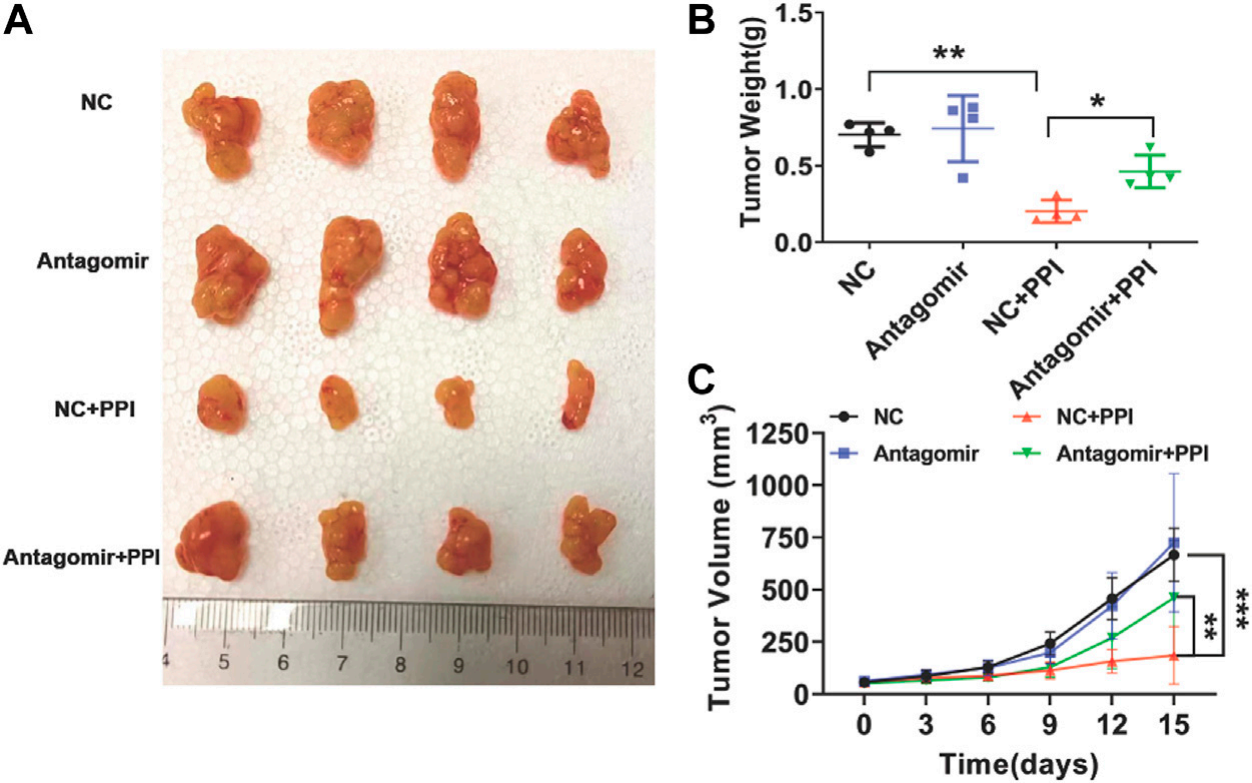
miR-124-3p is essential for PPI-mediated inhibition of the tumor growth *in vivo*. 1 × 10^6^ AGS tumor cells were injected *i.v*. to the nude mice. One week later, miR-124-3p antagonist (Antagomir) was injected to the tumors twice a week, and after the first injection, PPI or control vehicle was administered daily for up to 15 days. **(A)** Shown are photographs of tumors from PPI- and vehicle-treated mice injected with an antagonist of miR-124-3p (Antagomir) and negative control (NC) (n = 4 mice/group). **(B)** Tumors were weighed on day 15. **(C)** The volumes of the tumors were also detected at different timepoints. Data are displayed as mean ± SD (n = 4–6 mice/group, *p < 0.05, **p < 0.01, ***p < 0.001).

To further determine the involvement of miR-124-3p in tumor repression mediated by PPI *in vivo*, tumor-bearing nude mice were injected with an antagonist of miR-124-3p (Antagomir) and treated with PPI for up to 15 days. Here we found that PPI significantly reduced the tumor weights at day 15 compared with control (Figures 5A, B), whereas the suppression of miR-124-3p mostly reversed these inhibitory effects of PPI. Similar findings were also seen when tumor volumes were compared at different time points (Figure 5C). These findings confirmed an important role for miR-124-3p in PPI-induced inhibition of the cancer growth *in vivo*.

## Discussion

As a new type of regulated cell death, ferroptosis is characterized by the rapid accumulation of iron and iron-dependent lipid peroxidation (Dixon et al., 2012; Jiang et al., 2021; Tang et al., 2021; Fernandez-Garcia et al., 2022) Previous investigations have demonstrated that tumor cell ferroptosis is involved in cancer therapy (Wu et al., 2021). We have previously reported that PPI promotes cancer cell ferroptosis (Zheng et al., 2023). However, the molecular mechanisms underlying this effect of PPI remain largely unclear. In this study, we revealed that PPI increased miR-124-3p expression in both the gastric cancers *in vivo* and AGS/MKN-45 cells *in vitro*. More importantly, we demonstrated that miR-124-3p participated in PPI-induced cancer cell ferroptosis and suppression of the tumor growth.

Noncoding RNAs, including microRNAs, have been reported to modulate ferroptosis in cancer cells (Zhang H. et al., 2020a; Zhang X. et al., 2020b; Wei et al., 2021; Fuhrmann and Brune, 2022). On the other hand, induction of ferroptosis is desirable as a complimentary cancer therapy although it may contribute to an organ injury (Fuhrmann and Brune, 2022). We then examined if miR-124-3p is involved in the ferroptotic cancer cell death induced by PPI since previous studies have shown that miR-124-3p is adversely associated with tumor progression (Wu Q. et al., 2020a; Jin and Zhang, 2020; Li et al., 2021). We found that PPI upregulated the expression of miR-124-3p in gastric cancer cells *in vitro* and in the tumors *in vivo*, while inhibition of miR-124-3p in both cancer cell cultures and mice largely reversed the ferroptosis and suppression of tumor cell growth mediated by PPI. On the other hand, the mimics of miR-124-3p increased the gastric cancer cell ferroptosis, further confirming that PPI induces the ferroptotic cancer cell death through upregulation of miR-124-3p. The regulation of ferroptosis by miR-124-3p in the gastric cancer cells was likely carried out via directly targeting the 3′-UTR region of NRF2, resulting in the downregulation of NRF2 and subsequent ferroptosis of gastric cancer cells. NRF2 and its downstream signaling have been shown to regulate cell ferroptosis (Dodson et al., 2019). In our study, we demonstrated that PPI enhanced cancer ferroptosis by acting on miR-124-3p/NRF2 axis. Understanding of the mechanisms associated with ferroptotic cancer cell death could accelerate developing strategies for individualized cancer therapies. However, there is a limitation of our study in that our results still do not rule out a role for other microRNAs in gastric cancer cell ferroptosis. It’s also unknown whether miR-124-3P participates in cell ferroptosis of other types of cancers. Further investigations into these matters are warranted.

In conclusion, using two human tumor cell lines, we demonstrated that PPI augmented miR-124-3p expression in the gastric cancers *in vivo* and AGS/MKN-45 cells *in vitro*. We also found that miR-124-3p mediated the cancer cell ferroptosis and suppression of the cancer growth induced by PPI. Thus, these findings may help design new therapeutic strategies for treating human gastric cancers.

## Data Availability

The original contributions presented in the study are included in the article/Supplementary Material, further inquiries can be directed to the corresponding authors.
